# A non-invasive biomechanical device and treatment for patients following total hip arthroplasty: results of a 6-month pilot investigation

**DOI:** 10.1186/1749-799X-8-13

**Published:** 2013-05-21

**Authors:** Ganit Segal, Yaron Bar-Ziv, Steven Velkes, Vadim Benkovich, Gilad Stanger, Eytan M Debbi, Ronen Debi, Amit Mor, Avi Elbaz

**Affiliations:** 1 AposTherapy Research Group, Herzliya 46733, Israel; 2 Department of Orthopedic Surgery, Assaf Harofeh Medical Center, Zerifin 70300, Israel; 3 Department of Orthopedic Surgery, Rabin Medical Center, Petah Tikva 49100, Israel; 4 Department of Orthopedic Surgery, Soroka Medical Center, Beer Sheva 84101, Israel; 5 Department of Orthopedic Surgery, Barzilay Medical Center, 3rd Hahistadrut St., Ashkelon 78278, Israel

**Keywords:** Biomechanical therapy, Gait, Pain, Function

## Abstract

**Background:**

The purpose of the study was to examine the effect of a foot-worn biomechanical device on the clinical measurements and gait patterns of patients with total hip arthroplasty (THA).

**Methods:**

Nineteen patients, up to 3 months post-THA, were enrolled to the study. Patients underwent a computerized gait analysis to calculate spatiotemporal parameters and completed the Western Ontario and McMaster Universities osteoarthritis index and the SF-36 health survey. Patients then began therapy with a non-invasive foot-worn biomechanical device coupled with a treatment methodology (AposTherapy). Patients received exercise guidelines and used the device daily during their regular activities at their own environment. Follow-up examinations were conducted after 4, 12, and 26 weeks of therapy. Repeated measures ANOVA was used to evaluate changes over time. The clinical significance of the treatment effect was evaluated by computing the Cohen's effect sizes (ES statistic).

**Results:**

After 26 weeks of therapy, a significant improvement was seen in gait velocity (50.3%), involved step length (22.9%), and involved single limb support (16.5%). Additionally, a significant reduction in pain (85.4%) and improvement in function (81.1%) and quality of life (52.1%) were noted.

**Conclusions:**

Patients following THA demonstrated a significant improvement in gait parameters and in self-assessment evaluations of pain, function, and quality of life. We recommend further RCTs to examine the effect of this therapy compared to other rehabilitation modalities following THA and compared to healthy matched controls.

**Trial registration:**

Clinical trial registration number NCT01266382

## Background

Total hip arthroplasty (THA) is known to be a successful joint replacement procedure given that most patients experience significant pain alleviation, as well as an improvement in their health-related quality of life mostly during the first postoperative year and beyond [1,2]. The literature reveals, however, that despite these postoperative improvements, in some patients, the level of pain and the quality of life following THA do not reach those of the general population [1-3], nor does their gait pattern return to normal [4-6].

Gait analysis is a useful tool in the evaluation of locomotor function after THA [7]. Several studies have shown that joint motion does not return to normal after 6 months and in some cases up to years postoperatively [4,5,8]. This atypical joint motion includes additional stress being placed on the unaffected leg that may eventually lead to the development of osteoarthritis (OA) in the contralateral limb [6,9-11] and other joint disorders, some of which may even require a second arthroplasty [12]. However, other studies have reported that patients following THA do reach normal values. A recent study has examined the gait pattern outcome of two different surgical techniques for THA and reported one technique to be superior. Therefore, the surgical technique should be considered when evaluating the gait patterns of patients following THA. In addition, another study has examined resurfacing of the hip compared to THA and found similar results [13]. Both studies compared the operated patients with healthy controls and reported that the operated groups have reached the values of healthy controls.

In comparison with healthy individuals, THA patients generally exhibit a residual antalgic gait with slower gait velocity, shorter single limb support, and lower hip adduction and extension angles [14-18]. These deviations from normal individuals may be due to pain-avoidance strategies adopted preoperatively, apprehensions associated with the new prosthesis that leads to mechanical changes in the contralateral hip, and muscle weakness [4,5,14,15,19,20].

Gait adaptations to THA are also found at the non-operated hip and lead to limb asymmetry (i.e., differences in gait parameters between the operated and the non-operated limb) [21]. Patients with THA put less weight on their affected limb and do so less rapidly than with their unaffected limb [5,19,22-24].

A relatively new non-invasive foot-worn biomechanical device (AposTherapy) has been shown to improve clinical measures, as well as gait patterns and limb symmetry in patients with knee OA and chronic non-specific low back pain [25-29]. Specifically, this device was shown to reduce pain and functional limitation in patients with knee OA and to improve the quality of life of patients with knee OA. This therapy was also shown to improve gait velocity, step length, and single limb support of patients with knee OA and low back pain. Finally, the therapy was shown to improve kinematic and kinetic parameters of gait; mainly, it has been shown to reduce knee adduction moment. This therapy uses a device that has the capability of changing the location of the center of pressure (COP) during walking, thereby shifting the external forces acting on the body [30,31]. Furthermore, the device generates perturbation during movement that challenges neuromuscular control [32]. We presume that this therapy may have positive effect for patients following THA due to end-stage hip OA. The ability of the device to change the COP alongside controlled perturbation while walking will help patients following THA to comply with walking exercise and adopt new motion patterns. As mentioned, patients following THA present altered gait patterns compared to healthy controls, even a year post-surgery. We think that using this therapy will help return the gait patterns of these patients to be similar to the gait patterns of healthy controls.

The purpose of the current study was to conduct a pilot investigation to examine the applicability of this device on gait patterns, gait symmetry, and self-assessment levels of pain, function, and quality of life in patients following THA. We hypothesized that this therapy will lead to reduction in pain and improvements in the function and quality of life of these patients. We also hypothesized that these patients will show improved gait patterns and diminished limb asymmetry.

## Materials and methods

### Patients

Nineteen patients (ten males and nine females) with unilateral THA participated in this study. Their mean (SD) age was 63 (9.8) years, height was 164.6 (7.4) cm, and weight was 74.8 (15.9) kg. The inclusion criterion was a THA due to idiopathic hip OA up to 3 months post-surgery. Exclusion criteria were (a) previous THA in the contralateral hip, (b) other joint replacement procedures in the lower limbs, (c) pain or limitation in other lower extremity joints, (d) neurological and rheumatic inflammatory diseases, (e) postoperative dislocation or infection, (f) postoperative deep vein thrombosis, (g) malpositioning of the implant (abduction angle of the cup of 45° ± 10°), and (h) intraoperative periprosthesis fracture. All patients were implanted with cementless prostheses with ceramic on ceramic bearings (Corail and pinnacle cap, J&J, DePuy, Warsaw, IN, USA). All patients were operated with the same surgical technique. The surgical approach was modified in a translateral approach with splitting of the gluteus medius and minimus, opening the capsule and anterior dislocation of the hip joint. After reduction of the prosthesis components, the capsule was sutured as well as the gluteus medius and then the tensor fascia lata. In all patients, a subcutaneous drainage was used for the first 24 h. All patients were radiographically assessed immediately at the end of the operative procedure as well as 3 months post-surgery.

Informed written consent, approved by the Assaf Harofeh Medical Center ethic committee, was obtained from all patients prior to their involvement in the study (submission number 93/09, clinical trial registration number NCT01266382).

Patients were referred to the therapy center by a group of orthopedic surgeons between February 2009 and March 2010. All referred patients began therapy; however, some patients who were advised to begin this therapy declined to participate due to personal reasons (time-consuming, distance, did not want to commit). All patients underwent the traditional rehabilitation protocol as advised to them by their orthopedic surgeon prior to their arrival to the therapy center.

### Intervention

**Figure 1:**
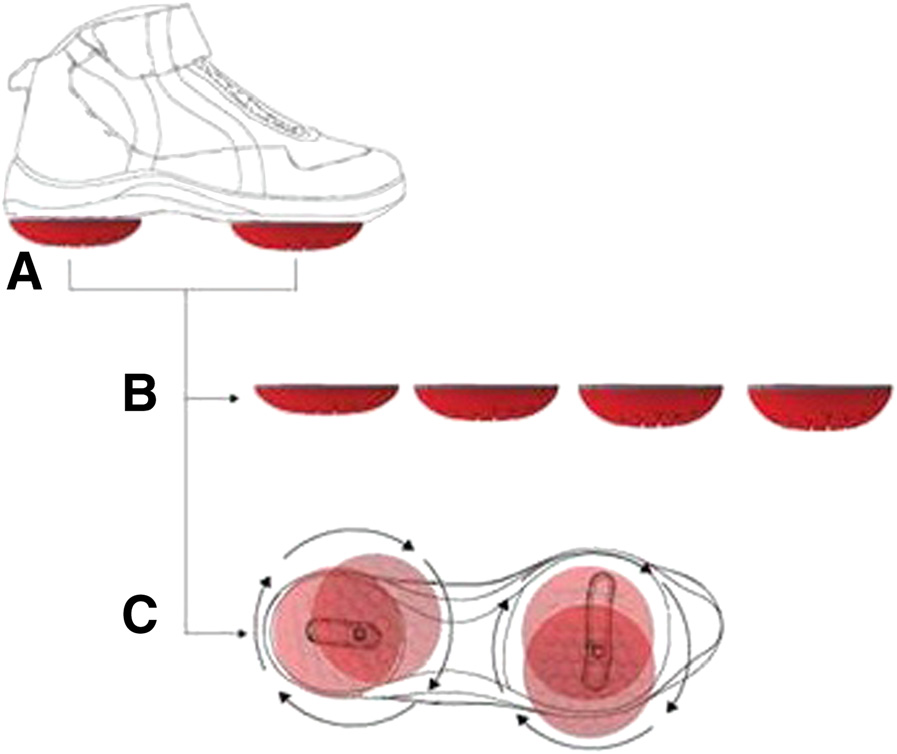
**Apos System.** (**A**) Biomechanical device comprising of two individually calibrated elements and a foot-worn platform. The elements are attached under the hindfoot and forefoot regions of the platform. (**B**) The biomechanical elements are available in different degrees of convexity and resilience. (**C**) The specially designed sole of the platform includes two mounting rails and a positioning matrix to enable flexible positioning of each biomechanical element.

The biomechanical system (Apos System, Apos–Sports and Medical Technologies Ltd., Herzliya, Israel) is a device combined with a treatment methodology. The device consists of four convex-shaped biomechanical elements with two attached to each of the patient's feet (Figure 1). One is located under the hindfoot region, and the other is located under the forefoot region. The elements are attached to the patient's foot using a platform in the form of a shoe. The platform is equipped with a specially designed sole that consists of two mounting rails that enable flexible positioning of each element under each region. The device is calibrated to the individual patient according to his or her pathology and motion characteristics. Each patient is asked to walk away from and then back towards the therapist. A visual gait evaluation is carried out by the therapist, and the device is appropriately calibrated. Appropriate calibration is defined as bringing the damaged joint to a biomechanical alignment that minimizes/eliminates pain. This is done by changing the location of the biomechanical elements, which changes the COP and shifts and changes the applied forces acting on the joints [30,31]. Together with biomechanical perturbations, this device enables home-based, dynamic, functional, and repetitive training intended to improve neuromuscular control. Following first assessment and calibration of the biomechanical device, each patient receives an exercise guideline according to a treatment protocol for each indication. In the current study, patients were instructed to follow a treatment protocol based on walking during activities of daily living, starting with 10 min of indoor walking each day during the first week and gradually increasing to 30 min of daily outdoor walking after 12 weeks.

### Questionnaires

All patients completed three questionnaires: (a) visual analog scale (VAS) for the operated hip and the non-operated hip, (b) the Western Ontario and McMaster Universities osteoarthritis index (WOMAC) [33], and (c) the Short Form Health Survey (SF-36) [34]. In addition, the therapist completed the Harris hip score (HHS) [35].

The average (SD) VAS score at baseline for the operated hip and for the non-operated hip was 28.4 (26.6) and 5.9 (11.9), respectively. The average (SD) WOMAC score of pain, stiffness, and function results at baseline were 17.1 (22.2), 20.6 (18.5), and 32.5 (19.1), respectively. The average (SD) score for the SF-36 physical scale and for the SF-36 mental scale at baseline were 49.3 (21.3) and 59.1 (21.6), respectively.

### Functional test

A timed up and go [36] (TUG) functional test was carried out. A standard height chair was used, and a line was placed 3 m from the chair. Each patient was asked to repeat the test three times, and the average of the three trials was calculated for further analysis.

### Gait analysis

The GaitMat system (E.Q. Inc., Chalfont, PA, USA) was used to measure spatiotemporal parameters [37]. During the gait test, all patients walked barefoot at a self-selected speed. Patients walked 3 m before and after the walkway mat (approximately 4 m in length) to allow sufficient acceleration and deceleration time outside the measurement area. Each gait test included four walks, and the mean value of the four walks was calculated for each parameter. Measured variables included: velocity (centimeter per second), step length (centimeter), and single limb support (SLS) (percent gait cycle, GC). SLS is a phase in the GC in which the body weight is entirely supported by one limb while the contralateral limb swings forward. Temporal distance (T-D) symmetry was calculated for SLS and step length using the formula:

$$\frac{\mathrm{involved}-\mathrm{uninvolved}}{\left(\mathrm{involved}+\mathrm{uninvolved}\right)/2}\times 100$$

A symmetry index value of zero represents perfect symmetry. A positive value indicates that the magnitude of that variable was larger in the involved limb, and a negative value denotes a larger magnitude in the uninvolved limb [38]. A 5% difference between limbs was considered to be a normal value.

### Protocol

All measured variables were evaluated during the first visit, after which the biomechanical device was individually calibrated. Treatment was then initiated and continued daily for a period of 6 months. Follow-up examinations were carried out after 4-, 12-, and at the 26-week end point. The physiotherapist verified compliance to the treatment protocol during each of the follow-up examinations.

### Statistical analysis

The goal of the proposed pilot study, which is one arm prospective study, was to test the hypothesis that there are significant improvements over time in pain, function, and SF scales. As this is a pre-experimental study, we could not determine the study sample size in advance. The criterion for significance (alpha) has been set at 0.050. The test is two-tailed, which means that an effect in either direction will be interpreted. With the proposed sample size of 19 patients and 19 for the two groups, the study will have a power of at least 80% to yield a statistically significant result. This computation assumes that the mean difference is 14.0 (corresponding to means of 17.0 versus 3.0), and the common within-group standard deviation is 14.3 (based on SD estimates of 20.0 and 3.0).

Data were analyzed with SPSS software version 19.0. (SPSS Inc., Chicago, IL, USA). The significance levels were set at 0.05.

The distributions of gait characteristics were examined using the Kolmogorov–Smirnov non-parametric test. Data were presented as mean and standard deviation for continuous variables following with 95% confidence intervals for the mean.

Changes over time for all measurements were calculated by the repeated measures ANOVA. The clinical significance of the treatment effect was evaluated by computing the Cohen's effect sizes (ES statistic, difference between two time points divided by the standard deviation at baseline) on gait velocity, step length, pain, and function and were compared to previous findings by other researchers.

## Results

None of the patients had postoperative complications including dislocation or infection as well as deep vein thrombosis. In addition, none of the patients had radiolucencies or suspected osteolysis. All patients complied with the treatment protocol. Compliance was verified at several points during the study. Patients received a telephone call to verify compliance at the 1st, 2nd, 10th, 16th, and 20th week of treatment. There were no reports of imbalance, tripping, or any other adverse events during the study period.

**Figure 2:**
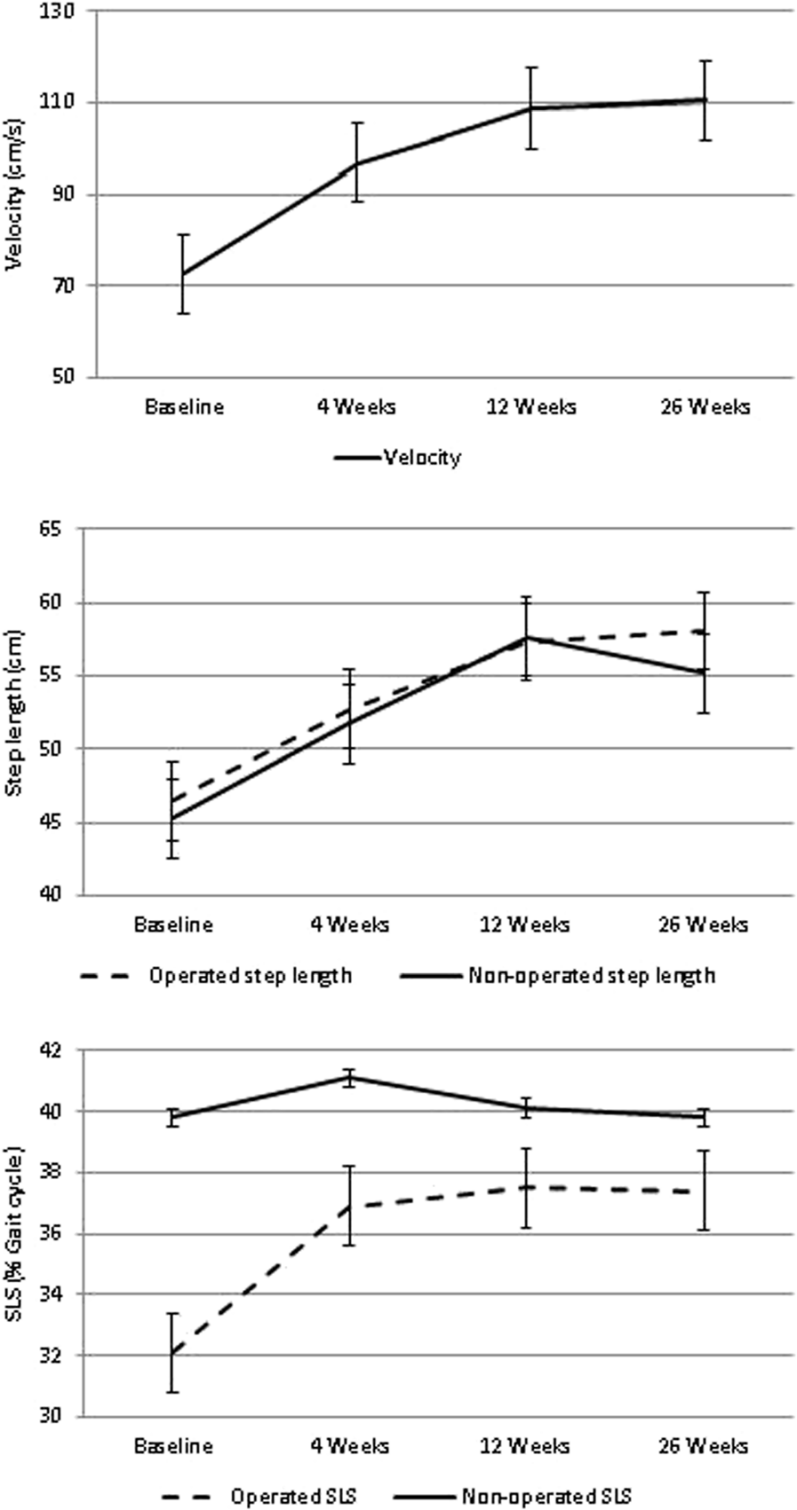
**Changes in gait velocity, step length, and single limb support following 6 months of AposTherapy.** A significant improvement was seen in all gait variables. All *P* values were <0.05. SLS, single limb support; GC, gait cycle.

All gait parameters (velocity, step length, and single limb support) improved significantly (*P* value ranged between 0.04 and <0.001) except for the uninvolved SLS. Significant improvements were found between all time points (4, 12, and 26 weeks), each time point compared to baseline, in all measurements except for the uninvolved step length at the 6-month time point (Figure 2). Step length symmetry and SLS symmetry significantly changed over time, each time point compared to baseline (*P* < 0.001). At baseline, there was a 17.4% asymmetry in step length and a −98.2% asymmetry in SLS. Step length asymmetry was 2.5%, 1.1%, and 10% after 4, 12, and 26 weeks of treatment, respectively. SLS asymmetry was −11.9%, −6.9%, and −6.8% after 4, 12, and 26 weeks of treatment, respectively. Alongside the improvement in gait measurements, a significant improvement was also seen in the TUG functional test between all time points, *P* < 0.001. Mean TUG (SD) was 12.6 (4.0) s, 8.1 (3.1) s, 7.6 (3.2) s, and 6.9 (2.7) s at baseline, 4, 12, and 26 weeks, respectively, (each time point compared to baseline). Overall, there was a mean improvement of 42% in the TUG test compared to the baseline result.

Patients reported a significant decrease in their level of pain (*P* value ranged between 0.05 and <0.001) and stiffness (*P* value ranged between 0.05 and <0.001) and a significant improvement in their level of function (*P* value ranged between 0.002 and <0.001) at all time points compared to their baseline results. Patients also reported a significant improvement in the SF-36 physical scale (*P* value ranged between 0.003 and <0.001) and mental scale (*P* value ranged between 0.002 and <0.001) compared to their baseline results (Table 1). When comparing the level of pain between the operated limb and the non-operated limb, based on the separated VAS scale for each limb, it was found that the level of pain in the operated limb has significantly reduced following the treatment whereas the level of pain in the non-operated limb did not change significantly over time (Table 2). The results of the HHS are summarized in Table 2. A significant increase was seen between all time points compared to their baseline results.

**Table 1:** WOMAC index and SF-36 Health Survey scores following 6 months of treatment

	**Baseline**			**4 weeks**			**12 weeks**			**26 weeks**			
	**Mean**	**95% CI**		**Mean**	**95% CI**		**Mean**	**95% CI**		**Mean**	**95% CI**		***P *value**^**a**^
		**Lower bound**	**Upper bound**		**Lower bound**	**Upper bound**		**Lower bound**	**Upper bound**		**Lower bound**	**Upper bound**	
WOMAC index (0–100)													
Pain	17.1 (22.2)	6.1	28.1	7.9 (12.2)	1.8	13.9	5.1 (8.0)	1.1	9.1	2.5 (2.9)	1.0	3.9	*P* = 0.007
Stiffness	20.6 (18.5)	11.4	29.9	12.6 (16.4)	4.4	20.7	8.0 (14.9)	0.6	15.4	3.9 (6.7)	0.5	7.2	*P* = 0.006
Function	32.5 (19.1)	23.0	42.0	13.4 (15.0)	6.0	20.9	10.9 (14.7)	3.6	18.2	7.8 (11.4)	2.1	13.4	*P* < 0.001
SF-36 Health Survey (0–100)													
Physical scale	49.3 (21.3)	39.0	59.5	59.9 (22.3)	49.2	70.7	69.6 (19.0)	60.4	78.7	75.0 (16.6)	67.0	83.1	*P* < 0.001
Mental scale	59.1 (21.6)	48.7	69.6	71.1 (18.5)	62.2	80.0	81.4 (12.6)	75.3	87.4	83.2 (15.3)	75.8	90.5	*P* < 0.001

^a^*P* value was set to *P* < 0.05. Results are presented as mean (SD) and 95% confidence interval (CI).

**Table 2:** VAS and Hariss hip form scores following 6 months of treatment

	**Baseline**			**4 weeks**			**12 weeks**			**26 weeks**			
	**Mean**	**95% CI**		**Mean**	**95% CI**		**Mean**	**95% CI**		**Mean**	**95% CI**		***P *value**^**a**^
		**Lower bound**	**Upper bound**		**Lower bound**	**Upper bound**		**Lower bound**	**Upper bound**		**Lower bound**	**Upper bound**	
VAS (0–10)													
Operated limb	28.4 (26.6)	15.2	41.7	13.6 (13.7)	6.7	20.4	6.2 (1.2)	0.3	12.2	1.4 (0.4)	0	3.2	*P* = 0.002
Non-operated limb	5.9 (11.9)	0	11.8	1.7 (4.0)	0	3.7	2.1 (5.7)	0	4.9	4.3 (10.3)	0	9.4	*P* = 0.21
Harris hip form	63.8 (18.1)	55.1	72.6	80.8	73.6	88.0	84.6 (13.2)	78.3	91.0	88.3 (11.9)	82.6	94.1	*P* < 0.001

^a^*P* value was set to *P* < 0.05. Results are presented as mean (SD) and 95% confidence interval (CI).

## Discussion

To date, there is insufficient evidence regarding the effectiveness of rehabilitation modalities for patients following THA. It is known that the level of pain and the quality of life of these patients following THA do not reach those of the general population [1-3], nor does their gait pattern return to normal [4-6]. The present study was a pilot investigation to examine the applicability of this device on gait patterns, gait symmetry, and self-assessment levels of pain, function, and quality of life in patients following THA. It was found that gait patterns, including gait symmetry, significantly improved over time. Furthermore, a significant improvement was also found in the TUG functional test. Patients also reported a significant reduction in pain and improvement in function and quality of life.

The gait characteristics of patients from the current study were compared with normal gait values drawn from the literature. Patients from the current study had reached normal SLS values after 12 weeks of treatment. This finding indicates that patients were able to bear the entire body weight on both the operated hip and non-operated hip. Results regarding gait velocity, however, are controversial. Compared to some studies, patients from the current study did not reach normal gait velocity values, whereas compared to other studies, they did [7,39,40]. Based on the finding of Kyriazis and Rigas that characterized the spatiotemporal parameters of patients following THA, patients from the current study have equaled their gait velocity value with the gait velocity value of the general population. Patients with THA from the general population reached this value after 8–10 years post-surgery [7], whereas patients in the current study have reached this value after 6 months.

An important finding of this study was the improvement in limb symmetry. Shakoor et al. suggested that patients with unilateral THA for end-stage OA are at high risk for future progression of OA in other joints of the lower extremities, often requiring additional joint replacements [12]. This is probably because of prolonged abnormal gait adaptations over the years prior to surgery and can also be a result of gait alterations in response to pain or fear following surgery [41]. In response to surgery, patients may unload the operated limb and overload the contralateral limb. Symmetry will be achieved over time; however, it will be on the expense of the healthy limb. For example, following a THA, the reduction in loads in the operated limb will reduce the SLS of the limb while the increase in loads in the contralateral limb will increase the SLS of the limb, even to values above the normal. As the body aspires to symmetry, the patient will reduce his or her SLS values in the healthy limb towards the values in the operated limb. In the current study, patients reached step length symmetry after 4 weeks and SLS symmetry after 12 weeks of treatment. Symmetry was achieved through an increase in step length in the contralateral limb and an increase in SLS in the operated limb, both reaching normal values.

Patients in the current study reported a significant reduction in pain and stiffness and an improvement in function and quality of life. The effect of rehabilitation programs for THA on the self-reported measures of function is not clear. Some studies did not find significant differences between physiotherapy rehabilitation and the control group, while others present some evidence indicating a treatment benefit within a treatment group compared to control [42,43].

Since this study did not have a control group, the results were compared to the results of other treatment modalities that have been previously reported. A recent comprehensive review examined the effectiveness of physiotherapy exercise following THA. It was concluded that there is insufficient evidence to establish the effectiveness of physiotherapy following THA for OA [42].

We further searched the literature to compare the results of the current study with previous reports. Table 3 summarizes the papers that were included in this comparison [44-47]. A calculation of the effect size (difference between two time points divided by the standard deviation at baseline) was carried out on the parameters gait velocity, step length, pain, and function. The effect size of AposTherapy on gait velocity was similar to the effect of treadmill training and conventional physiotherapy and was higher than all other treatment modalities including low-intensity training, high-intensity training, home-based exercise, and center-based exercise. The effect size of AposTherapy on the involved step length was substantially higher than all other treatment modalities. Similarly, the effect size of the therapy on the levels of pain and function was substantially higher compared to other treatment modalities.

**Table 3:** Characteristics of the five trials included in the comparison

	**Time post-surgery**	**Number**	**Treatment duration**	**Groups**	**Age**	**Weight (kg)**	**Height (cm)**	**Measurements**
Galea et al. [41]	2 months	23	8 weeks	Center-based exercise (*n* = 11)	68.6 (9.7)	76.3 (14.4)	160 (10)	WOMAC osteoarthritis index, gait velocity, step length
				Home-based exercise (*n* = 12)	66.6 (7.9)	81.6 (20.3)	160 (10)	
Husby et al. [45]	1 week	24	4 weeks	Strength training (*n* = 12)	58 (5)	84.6 (11.2)	174 (9)	Step length
				Conventional rehabilitation (*n* = 12)	56 (8)	80.9 (18.4)	170 (11)	
Jan et al. [46]	6 years	53	12 weeks	High-intensity training (*n* = 13)	58.8 (12.9)	Missing	159.5 (7.6)	Gait velocity
				Low-intensity training (*n* = 13)	59.3 (10.3)		158.4 (4.6)	
				Control (*n* = 27)	57.0 (12.8)		163.0 (9.7)	
Hesse et al. [47]	3 weeks	79	10 days	Treadmill therapy (*n* = 39)	64.7 (13.1)	70.9 (14.4)	166.4 (8.9)	Harris hip score, velocity
				Control (*n* = 40)	65.5 (9.9)	72.7 (12.1)	166.6 (8.6)	
Current analysis	3 months	19	26 weeks	AposTherapy (*n* = 19)	63.0 (9.8)	74.8 (15.9)	164.6 (7.4)	WOMAC osteoarthritis index, gait velocity, step length, single limb support

This study had some limitations. First, this study did not have a control group. Although the results of this study were compared to previously reported findings of studies that have examined the effect of other treatment modalities, it would have been preferable to use a control group. Secondly, this study had a relatively small number of patients. Another limitation to this study is a lack of information regarding the patient's gait characteristics and self-evaluation assessment prior to surgery. This information could have helped characterize the study population. In addition, this study included patients 3 months post-surgery with relatively low values of pain and functional disability but with compromised gait characteristics. Information regarding patients' pain and function level prior surgery and immediately after surgery would have helped in further understanding the study population. We recommend that future studies examine the effect of AposTherapy in patients with THA in a randomized controlled clinical trial.

## Conclusions

This study was a pilot investigation to examine the applicability of this device on gait and clinical measurements of patients following THA. The results of this study showed promising outcomes including significant improvements in gait patterns, functional tests, and self-evaluation questionnaires; however, future RCT's are warranted. These RCT's should include a comparison of this therapy modality to other common modalities and also compare this therapy with a group of healthy controls. This will help determine and relate the improvement to the therapy.

## Competing interests

One or more of the authors have received or will receive benefits for personal or professional use from a commercial party related directly or indirectly to the subject of this article. Ronen Debi, Avi Elbaz, and Amit Mor hold shares in AposTherapy. Ganit Segal and Gilad Stanger are salaried employees of AposTherapy. Yaron Bar-Ziv, Steven Velkes, Vadim Benkovich, and Eytan Debbi are co-researchers in a number of studies. They do not receive and are not entitled to any financial compensation from AposTherapy.

## Authors’ contributions

GS conceived and designed the study and participated in data collection, revising the article, and in the final approval. YBZ, SV, VB, and RD participated in the data collection, revising the article, and final approval. GS - data collection, drafting the article, final approval. EMD took part in drafting the article and final approval. AM and AE participated in the conception and design of the study, revising the article, and final approval. All authors take full responsibility for the entire manuscript content, integrity of the data, and the accuracy of the data analysis. All authors read and approved the final manuscript.
